# Study protocol: effect of infection, Modic and inflammation on clinical outcomes in surgery for radiculopathy (EIMICOR)

**DOI:** 10.1186/s12883-021-02377-4

**Published:** 2021-09-29

**Authors:** Niek Djuric, Geraldine Lafeber, Sjoerd G. van Duinen, Sandra Bernards, Wilco C. Peul, Carmen L. A. Vleggeert-Lankamp

**Affiliations:** 1 Department of Neurosurgery, Leiden University Medical Center, Albinusdreef 2, 2300 RC Leiden, The Netherlands; 2 Department of Pathology, Leiden University Medical Center, Albinusdreef 2, 2300 RC Leiden, The Netherlands; 3 Department of Medical Microbiology, Leiden University Medical Center, Albinusdreef 2, 2300 RC Leiden, The Netherlands; 4 Haaglanden Medical Center and HAGA Teaching Hospital, The Hague, The Netherlands; 5 Spaarne Hospital, Haarlem/Hoofdorp, The Netherlands

**Keywords:** Disc herniation, Cervical, Lumbar, Infection, Inflammation, Macrophages, Modic changes, Clinical outcomes, Radiculopathy

## Abstract

**Background:**

Evidence indicates that inflammatory processes are involved in radicular pain as well as in resorption of herniated disc tissue. Furthermore there are indications that the presence of vertebral end plate pathology (Modic changes; MC) is associated with a negative effect on inflammation. It is hypothesized that in patients with MC, the (possibly bacterial induced) inflammation will be accompanied by pro inflammatory cytokines that worsen the outcome, and that in patients without MC, the inflammation is accompanied by cytokines that induce a resorption process to accelerate recovery.

**Methods:**

This prospective cohort study will include 160 lumbar and 160 cervical patients (total of 320), which are scheduled for surgery for either a lumbar or cervical herniated disc with ages between 18 and 75. The main and interaction effects of local bacterial infection (culture), inflammatory cells in disc material (immunohistology), MC (MRI), and blood biomarkers indicating inflammation or infection (blood sample evaluation) will be evaluated. Clinical parameters to be evaluated are leg pain on the 11 point NRS pain scale, Oswestry (lumbar spine) or Neck (cervical spine) Disability Index, Global Perceived Recovery, Womac Questionnaire, and medication status, at baseline, and after 6, 16, 26 and 52 weeks.

**Discussion:**

Gaining insight in the aetiology of pain and discomfort in radiculopathy caused by a herniated disc could lead to more effective management of patients. If the type of inflammatory cells shows to be of major influence on the rate of recovery, new immunomodulating treatment strategies can be developed to decrease the duration and intensity of symptoms. Moreover, identifying a beneficial inflammatory response in the disc through a biomarker in blood could lead to early identification of patients whose herniations will resorb spontaneously versus those that require surgery.

**Trial registration:**

prospectively enrolled at trialregister.nl, ID:NL8464.

**Supplementary Information:**

The online version contains supplementary material available at 10.1186/s12883-021-02377-4.

## Background

Radiculopathy is a clinical symptom that has its origin in irritation of the spinal nerve. In patients with a bulging or herniating disc, compression of the nerve is considered the main cause. In more recent studies on radiculopathy, inflammatory processes appear to have a bigger role than originally thought. It is hypothesized that the disruptive process in the area of the spinal nerve starts with micro traumata in the vertebral endplate or disruption of the annulus fibrosus. Disruption of the annulus or micro breakage of the endplate at the location where the annulus is attached, leads to exposure of the nucleus pulposus to the epidural space [1]. In the epidural space, the nucleus pulposus may not only cause compression of the nerve, but is also exposed to the systemic circulation. This makes the disc prone to neovascularisation and macrophage infiltration, which is often seen in cervical and lumbar discs [2, 3]. Macrophage infiltration of the extruded material could promote a foreign body response and thereby worsen the symptoms [4]. However, they can also help to resorb the herniated material and thus alleviate the symptoms. This discrepancy could be explained by alternative macrophage differentiation: Macrophages can differentiate in many distinctive phenotypes with diverse functions [5], but they are often dichotomized in M1 and M2 macrophages [6]. Pro-inflammatory M1 macrophages are characterized by expression of CD40, CD192 and CD86 [6–8], and produce pro-inflammatory cytokines such as interleukin (IL)-1β, IL-6, IL-8, Tumor necrosis factor (TNF)-α, [5], all of which have been associated with and exacerbation of pain symptoms [9]. In contrast, M2 macrophages express markers such as CD163 and CD206 [6, 7], produce cytokines such as IL-4 and IL-10, and are believed to initiate resorption of the herniated disc material, which results in an amelioration of radicular pain and improvement of clinical outcomes [5, 9].

In our previous work, we found that the impact of the presence of macrophages on clinical outcomes was dependent on the presence of Modic changes (MC), which are considered inflammatory vertebral endplate signal changes that occur frequently on cervical and lumbar endplates [10, 11]. We found that in patients with MC, a higher degree of macrophage infiltration, was accompanied by more radicular pain symptoms and less favourable clinical outcomes [4]. In addition, we also showed that in patients without MC, a higher degree of macrophage infiltration was associated with less radicular pain and a better clinical outcome [4]. Based on these findings, it is to be expected that patients with MC have a higher degree of M1 macrophages, which would be in line with their slower recovery, while in patients without MC, larger numbers of M2 are to be expected, thereby explaining their faster recovery.

A different possible scenario is that the exposure to the systemic circulation is accompanied by infiltration of *Propionibacterium acnes* or *Staphylococcus epidermidis*, both of which are opportunistic bacteria that were demonstrated in lumbar and cervical herniated disc material [12], and have been associated with MC [13]. In the herniated disc, they could induce a host immune response which increases inflammation and could aggravate radicular symptoms [14, 15].

For future perspectives, it is important to understand the interconnectivity and clinical relevance of inflammation, infection and MC. Until now, no studies have sought to explore the relationship of these three together and how their interconnectivity affects clinical outcomes. Therefore, we would like to investigate the interactions between MC, bacterial infection, inflammation, and their impact on the clinical symptoms of both sciatica and cervical radiculopathy patients. Explicating these mechanisms may lead to characterization of certain subgroups, of which some may benefit from antibiotic treatment, some from anti-inflammatory treatment, some from a conservative approach and some from (early) surgery. Hence, identifying these subgroups will allow future studies to focus on specific treatments for each subgroup. If this is accomplished, we could significantly decrease the disease burden of patients that suffer from herniated discs.

## Methods

### Objectives

#### Primary objective(s)

1. In a group of lumbar and cervical radiculopathy patients undergoing disc surgery, this study will determine the impact of bacterial infiltration of the disc on patient reported pain scores, in the presence or absence of MC on MRI and histological defined disc inflammation.

#### Secondary objective(s)

1. A secondary aim of this study is to assess whether patients that suffer from disc inflammation benefit more from anti-inflammatory drugs than those without inflammation.
2. Another secondary aim of this study is to further explore the inflammation process by characterizing different types of macrophages (M1 and M2).
3. At last this study aims to associate the presence of bacterial infection to the presence and type of MC.

**Fig. 1:**
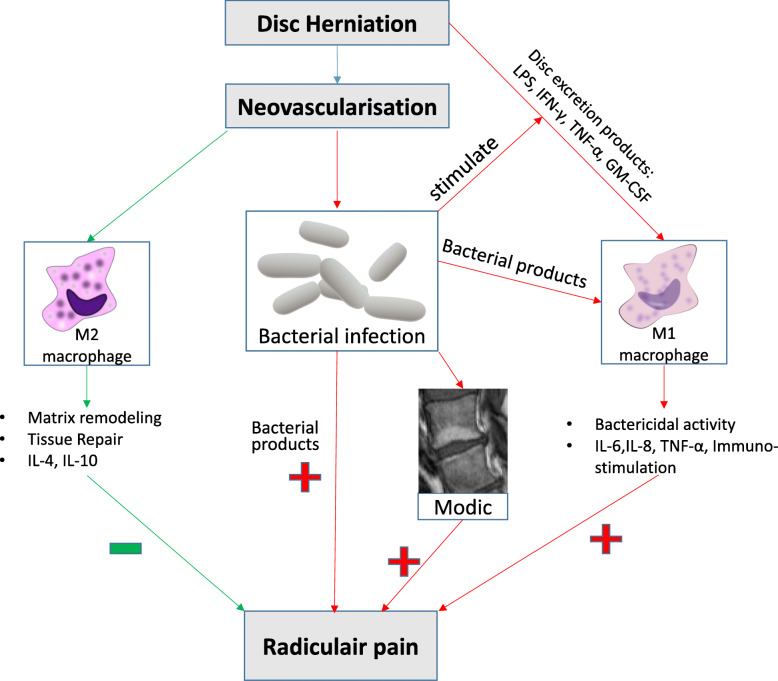
illustrates the hypothesis of the present study

Figure 1 illustration of the hypothesis:

After the disc is herniated, neovascularisation can be formed, which could lead to a couple of scenario’s. Firstly, in healthy discs, macrophages can enter and likely predominantly differentiate towards M2, which will help with disc resorption and reduce radicular pain. Secondly, likely in a degenerated disc, neovascularisation can be accompanied by infiltrating bacteria. By itself, this may irritate the nerve and cause pain symptoms, but it can also stimulate the disc to excrete pro inflammatory factors which worsen pain and may stimulate macrophages to differentiate towards M1. This could result in more M1 and less M2 macrophages and lead to more radicular pain symptoms. In addition, the adjacent endplate might also get involved, which could lead to more irritation of the adjacent nerve. Animations in the figure originate from cliparts.zone and are free to use, MRI picture is our own.

### Study design

This prospective-(longitudinal) observational cohort study is an imaging, histological, immunological and clinical study. Both the MRI scan and surgery are part of the usual care, in addition to these procedures, this study will draw 3 blood samples, use the rest material from surgery and ask the patient to fill in short pain related questionnaires.

The total duration of the study is approximately 2.5 years, of which 1.5 years for inclusion and 1 additional year for the follow up. Because Lumbar disc herniations occur more often than cervical ones, the inclusion of lumbar patients might be finished after 1 year, while the inclusion of the cervical patients will likely take 1.5 years. Patients will be recruited from one of the three inclusion centers in which they are planned for surgery: the Spaarne Gasthuis Haarlem Zuid, Alrijne Hospital Leiderdorp, HMC the Hague and Haga Hospital the Hague. The analyses and coordination of the study will be done from the Leiden University Medical Center (LUMC).

Patient recruitment will take place during the first (pre-operative) visit with the neurosurgeon, where the patient will be informed about the study. Patients that want to participate will be asked to sign an Informed Consent Form (ICF) in the week prior to the surgery. Afterwards, the participant will fill in a set of online questionnaires in Castor EDC. This set will contain questionnaires that assess demographic data, pain scores (NRS back/leg pain for lumbar patients and NRS neck/arm pain for cervical patients), disability scores (ODI for lumbar patients and NDI for cervical patients), Osteoarthritis (Womac) and medication status. Furthermore, the patient will receive an MRI scan in the weeks before surgery which is part of the usual care. In addition, during surgery, the herniated part of the disc will be dissected and transferred to the LUMC for further analyses. The dissected material will be used for three different purposes: One part of the dissected material will be used for bacterial culture, one part for histological analyses, and one part will be snap frozen for later defined analyses.. In addition to the frozen disc samples, this study will also collect blood samples for two purposes: the first is to assess the general inflammation (BSE, leukocyte differentiation) and vascular status (lipid and apolipoprotein profile) and the second purpose is for future analysis, for which a sample will be stored in the freezer. Only if our hypotheses turn out to be true, the stored blood samples will be used to search for a predictive biomarker for a bacterial infection and/or inflammation of the disc. The exact analysis that will be used to identify the biomarker will be defined in a later stage and will be based on our results from the histological analysis and bacterial cultures. Blood will be drawn in the waiting room for surgery from the canula that is placed for the purpose of the surgery. This means that only the donated blood is an additional aspect of the study, injecting the canula is part of the usual care.

**Fig. 2:**
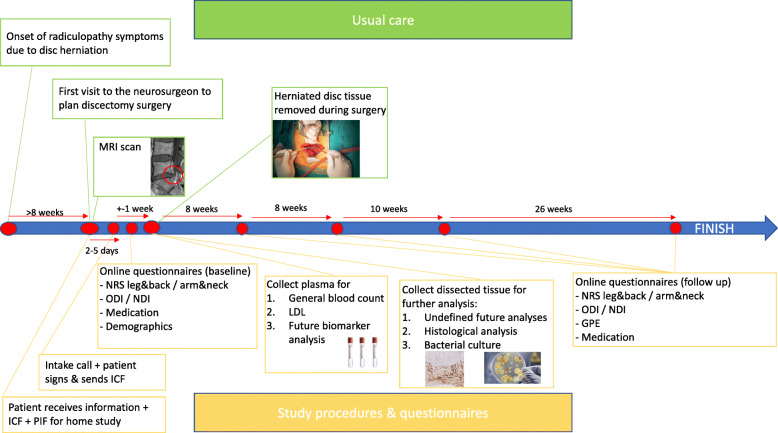
Flow chart of the EIMICOR study. MRI and histology images displayed in the figure are our own, the others were purchased and are now free of copyright

All participants will be asked to co-operate during the entire follow-up. During follow-up patients will be asked to fill in questionnaires regarding clinical outcome (NRS, ODI/NDI & GPE) and medication status at 8 weeks, 16 weeks, 26 weeks and 52 weeks post-surgery. Patients will receive emails with a link to the follow-up questionnaires at the above mentioned time points. A timeline of the study is provided in Fig. 2.

### Study population

#### Population (base)

For this study, all patients (18–75 yr.) with 8 or more weeks of radicular pain symptoms, that are eligible for surgery according to a neurosurgeon in the participating hospital, will be asked to participate. Eligibility criteria for surgery in participating hospitals are persisting pain symptoms after 8–12 weeks of conservative therapy with an MRI verified HNP that compresses the nerve root corresponding to the radicular pain symptoms. Patients are eligible for the study if they already planned to undergo surgery for herniated disc and meet the following in and exclusion criteria:

#### Inclusion criteria

##### Lumbar patients

- Age 18–75
- a unilateral lumbosacral radicular syndrome, with at least the following criteria:
  - o Radicular incitement: radiating pain from (a part of the) dermatome L4, L5 and/or S1
  - o Present for at least 8 weeks
- MRI verified lumbosacral disc herniation that is corresponding to the side of the symptoms
- Indication for surgery
- Informed consent

##### Cervical patients

- Age 18–75
- a unilateral cervical radicular syndrome, with at least the following criteria:
  - o Radicular incitement: radiating pain from (a part of the) dermatome C45, C56, C67 and/or C7T1
  - o Present for at least 8 weeks
- MRI verified cervical disc herniation that is corresponding to the side of the symptoms
- Indication for surgery
- Informed consent

#### Exclusion criteria

##### Lumbar

- Previous lumbar spinal surgery or chemonucleolysis
- Paresis of MRC < 4
- History of spinal inflammatory disease
- Instability that requires surgical fixation
- Active infection at the time of surgery
- Usage of Anti-biotics in the past six months
- Epidural steroid injection in the past six months
- Pregnancy
- Inadequate knowledge of the Dutch language

##### Cervical

- Previous cervical spinal surgery chemonucleolysis
- Paresis of MRC < 4
- Myelopathy as major complaint
- History of spinal inflammatory disease
- Instability that requires surgical fixation
- Active infection at the time of surgery
- Usage of Anti-biotics in the past six months
- Epidural steroid injection in the past six months
- Pregnancy
- Inadequate knowledge of the Dutch language

### Study parameters/endpoints

#### Main study parameter/endpoint

The main study parameter will be the NRS leg pain for lumbar patients and NRS arm pain for cervical patients. A description of these endpoints and all other parameters used during the study are described below.

##### NRS pain scores

- The pain experienced by the patients will be measured by questionnaires that assess leg pain for lumbar patients and arm pain for cervical patients: NRS leg pain, and NRS arm pain. In these validated questionnaires, the patients will display the amount of pain they have experienced in respective locations during the week previously to the visit. The pain intensity will be determined on a scale of 0–10. 0 represents ‘no pain’ and 10 represents ‘worst pain imaginable’ [16]. NRS will be focusing on multiple aspects of pain: Maximum/average intensity, frequency and maximum interference. All NRS pain scores will be measured during baseline (in the week before surgery) and at every follow-up moment (8, 16, 26, 52 weeks). Follow-up intervals 8, 26 and 52 were choosen based on our previous studies and 16 weeks was added as an extra time point in between [4]. Previous test results will not be visible for the patient. In addition to the NRS leg pain, NRS back pain will also be used as an additional outcome measure. Also, the NRS arm pain will be accompanies by the NRS neck pain as an additional outcome measure.

##### Other patient reported outcome parameters

- Functionality

For estimating functionality of the lumbar patient, the Oswestry Disability index (ODI) will be used. This validated questionnaire contains 10 topics related to the impact of the pain on the patient’s life, with 5 grading’s for each topic. The total will give a score between 0 (no disability) and 50 (maximum disability possible), which will be calculated to a 1–100% score [16]. For estimating functionality of the cervical patient, the Neck disability index (NDI) will be used, which is an adjusted version of the ODI focused on neck pain instead of back pain and is also a validated questionnaire. All functionality scores will be measured during baseline (in the week before surgery) and at every follow-up moment (8, 16, 26, 52 weeks,). Previous test results will not be visible for the patient.

- Recovery

In order to estimate the perceived recovery of the patients, the Global Perceived Effect (GPE) questionnaire will be used. The GPE is a widely validated questionnaire in which patients can express their perceived recovery on a 7 point Likert scale. On this scale the numbers 1–7 are accompanied by an expression of a state such as ‘Fully recovered’ = 7, ‘Same as before’= 4 or ‘Very bad’ = 1. All recovery scores will be measured at every follow-up moment (8, 16, 26, 52 weeks,). Previous test results will not be visible for the patient.

##### Predictive parameters

- Bacterial infection of the disc

Bacterial infection in the disc will be verified by a bacterial culture protocol. Tissue necessary for the bacterial culture will be harvested from herniated disc tissue that was taken out during surgery. The bacterial culture will be accompanied by a gram stain and methyl blue stain. Infection can be distinguished from a contamination by the quantity of colonies on the culture (0–10 is considered as contamination). Nevertheless, since it cannot be ruled out that the presence of 0–10 colonies is an infection instead of contamination, additional analysis with 0–10 regarded as infection will also be performed.

- Disc inflammation

Disc material harvested during surgery will be stained for the presence of macrophages, for M1 and M2 macrophages separately. Evaluation will be done through counting cells and subsequently categorizing them.

- Modic Changes

Type (1,2 or 3) and severity (< 50% & > 50%) of MC will be scored at baseline on MRI. Type 1 shows an hypointense endplate on T1 MRI and a hyperintense endplate on T2 MRI, Type 2 shows an hyperintense endplate on both T1 and T2 MRI and Type 3 shows a hypointense endplate on both T1 and T2 MRI.

#### Secondary study parameters/endpoints

A possible mediator in this study is the amount and type of pain medication that patients take, either in self-care or on prescription from the neurosurgeon or GP as part of the usual care. This could potentially alter the inflammation profile (in the case of NSAID’s) or could lead to lower perceived pain scores. In addition, it could be that patients with severe inflammation benefit more from anti-inflammatory drugs. Therefore this study will measure participants usage of pain medication as follows:

##### Pharmacological data

participants will be asked to fill in a form regarding the frequency, type and dosage of pain medication and anti-inflammatory drug usage. This form will be given to the participant at baseline (in week before surgery) and will also be given during the one-year follow-up at 8, 16, 26 and 52 weeks.

##### Osteoarthritis

In order to investigate to what extent MC are related to clinical features of osteoarthritis, participants will be asked to fill in the Womac questionnaire. The Western Ontario and McMaster Universities Osteoarthritis (Womac) index questionnaire evaluates pain and physical function, containing 24 questions about daily functioning and stiffness. The questionnaire will be given to participants at baseline and after 1 year follow-up.

#### Other study parameters

Furthermore, some additional study parameters that may cause confounding will be measured:

##### Demographic data

From all patients, general information will be collected: age, sex, BMI, ASA, smoking habits, duration of radiating pain symptoms and comorbidity including: hypercholesteremia, hypertension, chronic heart failure, myocardial infarction, angina pectoris, COPD, stroke and diabetes (not only as comorbidity, but also due to possible polyneuropathy symptoms). Furthermore, work-related risk factors will be assessed (professional driver, physical labour and/or heavy lifting during work).

##### MRI data

nerve root compression (no or mild, moderate, severe) will be scored on MRI (compression is believed to lead to more severe pain symptoms)

### Study procedures

#### Surgery techniques lumbar surgery

A unilateral transflaval discectomy will be performed according to usual care. Patients are placed in the knee-elbow position. Using anatomical landmarks and fluoroscopy the level of incision is determined. A small midline incision in the lumbosacral region is made. Muscles are unilateral detached from the spinous process. After spreading the wound a very small partial resection of the rostral lamen is executed, followed by a flavectomy with unilateral opening of the lateral recess. The nerve root is identified as well as the bulging disc. A discectomy is performed and the tissue taken out is assembled in a jar, identified with patient name and study number. If the nerve root is compressed by a sequester, only a sequesterectomy is performed if deemed necessary by the surgeon. After decompressing the nerve and performing a discectomy, the wound is closed in layers.

Surgery will be performed by a qualified neurosurgeon. Postoperative care will consist of an admission period of 2 days (day of surgery and day after) and one postoperative visit to the physiotherapist. From there on, the general practitioner will take care of the postoperative care.

#### Surgery techniques cervical surgery

For anterior discectomy, the level of surgery is verified by fluoroscopy. The operation will be carried out by a qualified neurosurgeon. Most surgeons operate using loupe magnification. The platysma muscle is separated or cleaved at the right side of the midline (less frequently on the left side), and the prevertebral space is reached by an approach medial to the sternocleidomastoid muscle and the carotid artery, and lateral to the trachea and oesophagus. The disc is incised and the corpora are distracted. Discectomy is performed as thorough as possible. Regularly the posterior ligament is cut and the spinal root is decompressed. If necessary, spondylarthrotic rims are removed. To the preference of the surgeon bone graft or an intervertebral fusion device is left behind.

#### MRI protocol

The MRI imaging process, which are part of the usual care, will be done according to the standard protocol of each participating hospital. Every patient will receive a series of images performed by a 9.0 Tesla scanner:

- Sagittal T1SE (turbo spin echo)
- Sagittal T2TSE
- Transversal T1-TSE
- Transversal T2-TSE

Evaluation of the MRI’s will be done by two independent researchers, both experienced in evaluating spine MRI scans, by using two evaluators, this study can perform a intra agreement analyses and provide a kappa value to put the accuracy of the evaluators in perspective. The evaluators will describe the disc characteristics (bulging, herniated or sequestrate) and the severity of nerve root compression. In addition, the images will be scored on the presence, severity and type of MC. The Type will be scored according to criteria from Modic et al. [17, 18]. Type 1 shows an hypointense endplate on T1 MRI and a hyperintense endplate on T2 MRI, Type 2 shows an hyperintense endplate on both T1 and T2 MRI and Type 3 shows a hypointense endplate on both T1 and T2 MRI. Severity of MC will be categorized as > 50 and < 50%.

#### Disc sample

During surgery, the neurosurgeon will collect a sample of the herniated disc tissue that was removed during the procedure, which will be used for further histological analysis.

All harvested discs will be fixed in a 4% formaldehyde solution for 3–7 days. Tissue will subsequently embedded in paraffin blocks and 5-μm thick slices shall be taken from the middle of the block for haematoxylin staining. Stained coupes will be evaluated under the microscope for clear signs of infiltrating inflammatory cells, if tissue from one sample exceeded the capacity of 1 paraffin block, multiple blocks will be formed and a slide of each block will be evaluated. One slide of each disc that contained inflammatory cells was submitted to further analysis using immunohistochemistry:

Presence of M1 macrophages will be characterized by the co-presence of CD68 (DAKO, Denmark), with CD192 (Thermofisher,U.S.A) [6]. Presence of M2 macrophages on the other hand, will be verified by co-presence CD68 and CD163 (Abcam, Netherlands). In order to be certain that the selected anti-bodies are a valid tool to assess the presence of M1 and M2 macrophages, the panel has been tested in a pilot study In this pilot study, T-cells (CD3) and Neutrophils (CD15) were present in very low quantities and hence not included in the study protocol.

For the staining procedure, 5-μm paraffin slices will be rinsed in ethanol and methanol solutions and prepared for the expression of CD68, CD192, CD163. Immunohistochemistry will be performed using a three-step indirect method. Antibodies will be cooked in EDTA pH 9.0 buffer as a pre-treatment. Subsequently, an avidin-biotin complex technique will be performed with the Vectastain ABC-Elite Kit (Vector Lab. USA) and the appropriate biotinylated antibodies. Visualization of the peroxidase reaction will be done with DAB solution (Sigma). Moreover, samples will be counterstained with Harris haematoxylin. All of these samples will be accompanied by a positive control. In control samples, primary antibodies will be omitted, which results in the expected absence of any cellular labelling. In order to standardize the evaluation of the samples, all samples were photographed under the microscope before they were evaluated. Since our pilot study showed that CD68 and CD192 was also expressed by nucleus pulposus cells/chondrocytes, cells were analysed based on morphological features and only macrophages were photographed and evaluated. The same approach was used for CD163.

In order to assess and characterize the bacterial infiltration of the herniated discs, additional samples will be extracted from the resected tissues. Disc samples will be used for anaerobic and aerobic bacterial cultivation, gram staining and methyl blue staining, to verify and characterize a bacterial infection according to standard clinical protocol. Samples with > 10 colonies per species per culture will be scored as infection, In contrast, samples with 0–10 colonies per species will be scored as contamination. At last, a third disc sample will be collected from the herniated excised tissue, and will be snap frozen for future analysis.

#### Blood samples

Blood samples will be collected from the canula directly after it has been inserted on the holding (OR preparation room). By doing so, the blood sample for the study will be drawn before the patient receives prophylactic antibiotic treatment according to the usual care. A total of 3 blood samples will be collected: the first sample (4 ml EDTA) will be used for a general blood count, the second (3 ml Heparin) for lipid and apolipoprotein profiling, these samples will be send directly to the laboratory in respective hospitals, the 3th and 4th samples will be collected in a 4 ml EDTA for future purposes and will be transferred to the LUMC at the end of the day. At the LUMC, the 3th sample will be deposited at the microbiology department where the plasma will be subtracted according to standard protocol and stored in the freezer. Since the plasma samples will only be analyzed if the hypotheses turn out to be true, the exact analysis protocol will be defined at a later stage.

### Statistical analysis

#### Sample size calculation

**Fig. 3:**
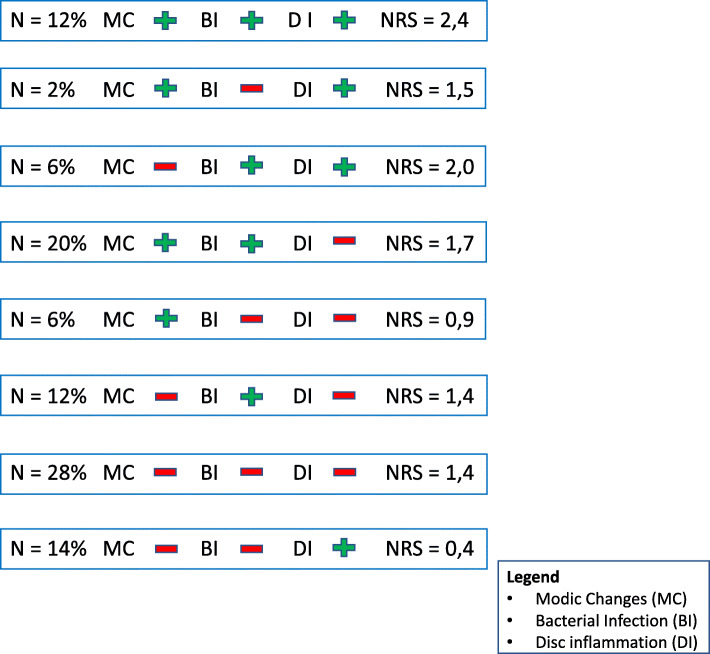
Subgroups with hypothesized average NRS scores during the one year follow-up

Distinguishing between the presence of MC (yes/no), bacterial infection (yes/no) and inflammation (yes/no) we have 2x2x2 = 8 groups. We expect the mean during follow-up of the primary outcome (NRS pain score) and the distribution of patients among the 8 groups to be as in Fig. 3. The standard deviation of the NRS is expected to be 1. These expectations were based on findings from our earlier study that described the interaction effect between MC and inflammation on the clinical outcomes [4], the impact of the presence of bacteria was estimated based on findings from Dudli et al. 2018, which showed that the pro inflammatory reaction to bacteria in the disc depends on the presence of MC [15]. We will primarily test for *any* effect of bacterial infection by comparing the full factorial model to a model without bacterial infection by means of an F-test on 4 degrees of freedom. We implemented a Monte-Carlo simulation in the statistical software R to compute the appropriate sample size. We found that a total of 160 patients suffices to have about 90% power to detect any effect of bacterial infection (Supplementary Figure S1). Since these numbers will also suffice for the secondary goals of the study, we intend to include 160 lumbar and 160 cervical subjects. Since the study burden is low for the participants, we expect a low drop-out rate of + − 10%.

For the statistical analysis, a linear mixed model will be used in which bacterial infection, Modic Changes and disc inflammation are used as a fixed factor and the NRS-scores of all time points (baseline, 8, 16, 26, 52 weeks) are used as an outcome measure. The model will be full factorial (assess all main and interaction effects). In addition, age, sex, nerve root compression and pain medication will be considered as a covariate.

All secondary study parameters will be analysed by multiple tests including a Pearson/Spearman correlation test, linear mixed model and a Chi-square test for categorical variables.

## Discussion

At present, the guideline for radiculopathy is the same for all patients: a wait and see approach, and surgery is only offered to those with persevering symptoms. Even after surgery, for some patients the symptoms persist or return after a short period of relief. The great variety in how radiculopathy patients recover indicates that our ‘one size fits all’ model for treating radiculopathy requires refinement. Previous research has already indicated that inflammatory cells such as macrophages play a crucial role in recovery and that the extend of this inflammation response varies from patient to patient [2]. Moreover, the presence of inflammation is not always a beneficial sign, as recent studies have indicated that presence of MC seem to indicate a chronically irritating inflammation response [4], and others have found bacteria in herniated discs [14]. Such findings strongly indicate that refinement of our current treatment approach is needed and that radiculopathy patients should be further sub characterized based on their inflammation status. Up till now, the evidence for inflammation subgroups within radiculopathy is not convincing and most studies focus on only one aspect without incorporating the rest of the inflammation status. For example, many recent studies have focussed on proving bacterial presence in herniated discs, but none have assessed whether this has any impact on clinical outcomes [14]. Other studies have tried to treat patients with anti-inflammatory drugs but failed to assess whether inflammation was present in these patients [19, 20]. Some studies have associated presence of MC with poor clinical outcome but failed to incorporate inflammation status [21]. Further, many studies required information regarding the inflammation status from analysing the disc material from surgery and don’t look for biomarkers in blood [22–25]. Others focus on drawing blood without verifying whether the blood results correspond to the status of the disc [26–28]. In order to create a more personalized treatment approach it is first essential to understand which patients are affected by what kind of inflammation and how this affects clinical outcomes. Moreover, it is important to explore biomarkers in blood that reliably resemble these different inflammation statuses, so personalized clinical decisions can be taken in an early disease stage without invasive and costly diagnostic procedures. Therefore, the EIMICOR will be the first study to incorporate all various types of data on tissue and blood level and assess their relevance concerning clinical outcome. By doing so we aim to connect the dots and elucidate the complex role of inflammation in sciatica.

### Possible operational issues

The design of the present study is aimed at reducing the amount of time and effort that is required from the participants and surgeons as much as possible. At the same time we focus on saving unnecessary costs by postponing additional analyses until our initial hypothesis is confirmed and additional analysis are more likely to reveal critical information. Unfortunately, this design comes with some practical issues that have to be dealt with. For instance, transferring the samples to the LUMC takes time and if surgery is scheduled late in the afternoon, it could mean that the samples cannot be processed in the LUMC the same day which may impact the quality of the data. Therefore it is crucial for the quality of our data that the inclusions are tailored to the OR schedules.

Moreover, as participants fill in the follow-up questionnaires through an email link, it makes it harder to control if they fill in the questionnaires, which may lead to missing data. In order to manage this issue, the status of the questionnaire will be monitored frequently and participants that forgot to fill in the questionnaires will be reminded to do so by email and phone.

## Supplementary Information

**Additional file 1: Figure S1.** Monte Carlo simulation for sample size calculation.

## Data Availability

Data and materials will be stored for 15 years after study completion. All data generated or analysed during this study will be included in the published article [and its supplementary information files].

## Abbreviations

MC: Modic Changes
LPS: Lipopolysaccharide
IFN-γ: Interferon-gamma
TNF: Tumor necrosis factor (TNF), and expression of
GM-CSF: Granulocyte macrophage colony-stimulating factor
M-CSF: Macrophage colony-stimulating factor
IL: Interleukin
ICF: Informed consent form
NRS: Numerical Rating Scale
ODI: Oswestry Disability Index
NDI: Neck Disability Index GPE Global Perceived Effect
LUMC: Leiden University Medical Center
