# Supplementary material for: Study protocol: effect of infection, Modic and inflammation on clinical outcomes in surgery for radiculopathy (EIMICOR)
Source: BMC Neurol. 2021 Sep 29;21:379. doi: 10.1186/s12883-021-02377-4 (PMC8480036; doi:10.1186/s12883-021-02377-4)
Supplement: Supplementary file 1 — Additional file 1: Figure S1. Monte Carlo simulation for sample size calculation. [file 12883_2021_2377_MOESM1_ESM.docx]

**Supplementary Appendix:**

**Figure S1: Monte Carlo simulation for sample size calculation**

Figure S1 displays the results of the Monte Carlo simulation for calculating sample size. The Y axis displays the statistical power that corresponds to a samples size on the X-axis. The largest difference is expected between the groups DI+/BI+/MC+ vs DI-/BI-/MC-, for which 160 patients will results in a statistical power of 90%. With this sample size, differences between the other subgroups (purple, blue and green lines), will not reach sufficient statistical power.

DI = Disc inflammation, BI = bacterial infection, MC = Modic changes.
